# Synthesis of 20‐Membered Macrocyclic Pseudo‐Natural Products Yields Inducers of LC3 Lipidation

**DOI:** 10.1002/anie.202114328

**Published:** 2022-01-25

**Authors:** Georg Niggemeyer, Anastasia Knyazeva, Raphael Gasper, Dale Corkery, Pia Bodenbinder, Julian J. Holstein, Sonja Sievers, Yao‐Wen Wu, Herbert Waldmann

**Affiliations:** ^1^ Max Planck Institute of Molecular Physiology Department of Chemical Biology Otto-Hahn-Strasse 11 44227 Dortmund Germany; ^2^ Technical University Dortmund Faculty of Chemistry, Chemical Biology Otto-Hahn-Strasse 6 44221 Dortmund Germany; ^3^ Umeå University Department of Chemistry 90187 Umeå Sweden; ^4^ Umeå University Umeå Center for Microbial Research 90187 Umeå Sweden; ^5^ Max Planck Institute of Molecular Physiology Crystallography and Biophysics Unit Otto-Hahn-Strasse 11 44227 Dortmund Germany; ^6^ Technical University Dortmund Faculty of Chemistry, Inorganic Chemistry Otto-Hahn-Strasse 6 44221 Dortmund Germany; ^7^ Compound Management and Screening Center (COMAS) Otto-Hahn-Strasse 11 44221 Dortmund Germany

**Keywords:** Autophagy, Bioorganic Chemistry, Macrocycles, Natural Products, Pseudo-natural Products

## Abstract

Design and synthesis of pseudo‐natural products (PNPs) through recombination of natural product (NP) fragments in unprecedented arrangements enables the discovery of novel biologically relevant chemical matter. With a view to wider coverage of NP‐inspired chemical and biological space, we describe the combination of this principle with macrocycle formation. PNP‐macrocycles were synthesized efficiently in a stereoselective one‐pot procedure including the 1,3‐dipolar cycloadditions of different dipolarophiles with dimeric cinchona alkaloid‐derived azomethine ylides formed in situ. The 20‐membered bis‐cycloadducts embody 18 stereocenters and an additional fragment‐sized NP‐structure. After further functionalization, a collection of 163 macrocyclic PNPs was obtained. Biological investigation revealed potent inducers of the lipidation of the microtubule associated protein 1 light chain 3 (LC3) protein, which plays a prominent role in various autophagy‐related processes.

## Graphical abstract

The combination of three natural product fragments in 20‐membered macrocyclic pseudo‐natural products led to the discovery of inducers of a novel LC3 lipidation phenotype.

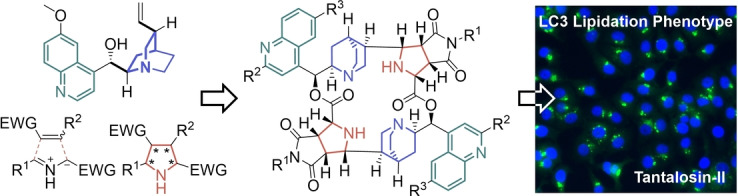

## Introduction

The structures of natural products (NPs) have inspired a wealth of research programs aimed at the discovery of new compound classes endowed with biological activity, and NPs are a rich source of drugs.^[1]^ In light of this long‐standing success, novel principles for the design and synthesis of NP‐inspired compound classes are in high demand. For wider exploration of NP‐like chemical space we have recently introduced the pseudo‐natural product (PNP) principle.[^2^, ^3^, ^4^, ^5^]

In PNPs natural product derived fragments are combined in unprecedented arrangements currently not accessible by known biosynthesis pathways.^[6]^ They define novel classes of biologically relevant compounds with activities that differ from their parent NPs.^[7]^ Thus, for instance PNPs that inhibit glucose uptake,^[8]^ cytokinesis^[9]^ or autophagy^[10]^ have been identified.

For PNP design and synthesis, different guidelines and strategies have been devised..[^4^, ^5^] However, wider exploration of chemical and biological space could be feasible if the PNP concept would be combined with alternative design principles. Thus, we have recently shown that de novo fragment combination can advantageously be combined with Hergenrother's ring‐distortion complexity‐to‐diversity strategy.^[11]^

In a further reaching design approach, NP fragments were incorporated into macrocyclic peptides to yield conformationally diverse macrocyclic peptide‐NP‐scaffold hybrids which were termed “PepNats”. These macrocycles mimic “hot loop” peptide sequences involved in protein‐protein interactions^[12]^ and yielded novel selective ligands for two different target proteins.^[13]^

**Scheme 1:**
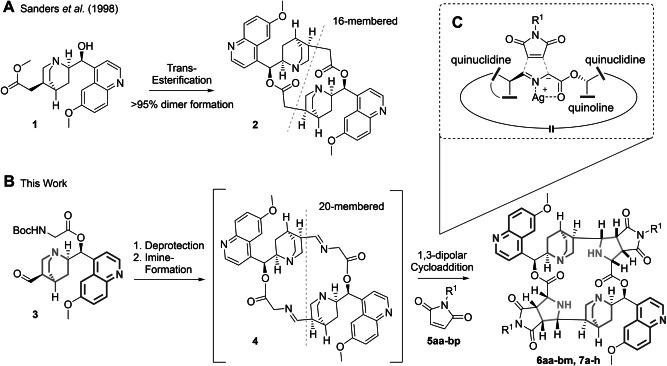
Strategy for the synthesis of macrocyclic pseudo‐NPs composed of two cinchona alkaloid derived fragments and two pyrrolidines. A) Formation of homodimeric quinidine macrolactone **2** by transesterification.^[17]^ B) Formation of homodimeric dual imine intermediate macrocyclic intermediate **4** and conversion into macrocycles **6** and **7**. C) Possible transition state of the planned 1,3‐dipolar cycloaddition on one hemisphere of the macrocycle. Grey dashed line represents C_2_‐symmetry.

PepNat development explores the fact that macrocycles, due to their structural preorganization and size, in general enable targeting of larger, often flat protein surfaces frequently involved in protein–protein interactions.[^14^, ^15^, ^16^] Thus, in order to further expand PNP chemical space, we sought to design novel non‐peptidic PNP types, which both incorporate different NP fragments and are macrocyclic. For the efficient synthesis of such NP‐derived macrocycles, we were inspired by a finding of Rowan and Sanders et al., who had observed that transesterification of (fragment‐sized) quinidine alkaloid methyl ester **1** nearly exclusively yielded macrocyclic homodimer **2**, due to a preorienting structural templating effect and thermodynamic equilibration (Scheme 1A).[^17^, ^18^] We envisioned to exploit such a templating effect and employ it in a more general sense to generate cinchona‐alkaloid‐containing homodimers, which then could be subjected in situ to stereoselective transformations resulting in the formation of additional fragment‐sized NP structures within the preformed macrocycles (Scheme 1B). Dimerization can be an efficient tool to quickly build up complexity and is estimated to be found in the biosynthesis of 15–20 % of all NPs.^[19]^

**Scheme 2:**
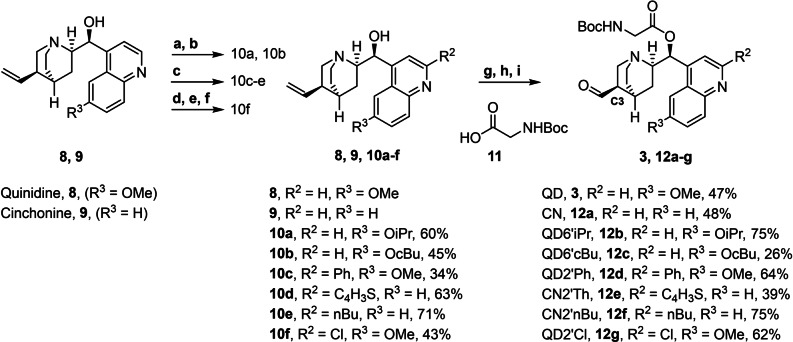
Synthesis of cinchona alkaloid‐derived aldehydes **3** and **12 a**–**g**. a) BBr_4_ then NH_3_ aq. (78 %); b) alkyl halide, Cs_2_CO_3_ (57–77 %); c) organolithium then MnO_2_ (34–71 %); d) *m*CPBA then e) H_2_SO_3_ aq. (97 %); f) POCl_3_ then NH_3_ aq. (44 %); g) Boc‐Gly‐OH, DCC, DMAP (78–96 %); h) OsO_4_, K_3_[Fe(CN)_6_], K_2_CO_3_, H_2_O (35–91 %); i) NaIO_4_, SiO_2_ (84–93 %).

In particular, it was planned to convert the exocyclic double bond on the quinuclidine ring into an aldehyde and to esterify the secondary benzylic alcohol with a Boc‐protected amino acid (see **3**, Scheme 1B and Scheme 2). The deprotected, bifunctional derivative obtained from **3** could then reversibly form macrocyclic dimeric Schiff base **4**. Azomethine ylides formed in situ by deprotonation from these amino acid ester imines could subsequently undergo stereoselective 1,3‐dipolar cycloadditions with diverse olefins (Scheme 1C).[^20^, ^21^, ^22^, ^23^] Thereby novel macrocycles **6** and **7** would be formed with embedded pyrrolidine fragments which are themselves the defining scaffold of numerous natural products.[^24^, ^25^, ^26^] If both hemispherical cycloadditions follow the same stereochemical path, the resulting dimers will be C_2_‐symmetrical.

Here we describe the implementation of this strategy in the synthesis of 20‐membered macrocyclic PNPs composed of two cinchona‐alkaloid fragments and two pyrrolidine fragments. Biological investigation of these novel macrocyclic PNPs led to the identification of potent upregulators of the lipidation of the microtubule associated protein 1 light chain 3 (LC3) protein, which plays a prominent role in various autophagy‐related processes.

## Results and Discussion

In order to establish the envisioned macrocyclization sequence, quinidine analogs **10 a**–**f** carrying different substituents in the quinoline ring (synthesized as described by Hintermann et al.,^[27]^ Shiomi et al.^[28]^ and Wang et al.;^[29]^ for details see below) were esterified with a Boc‐protected glycine and subsequently converted into aldehydes **3** and **12 a**–**g** by means of Sharpless dihydroxylation followed by diol oxidation with NaIO_4_ (Scheme 2).^[30]^

After removal of the Boc group with TFA, exchange of solvents and addition of organic base, the resulting amino‐aldehydes rapidly formed macrocyclic dimeric Schiff bases analogous to **4**, as was shown by means of reduction via NaBH_3_CN and subsequent HPLC‐MS analysis (see Supporting Figure 1).

**Figure 1:**
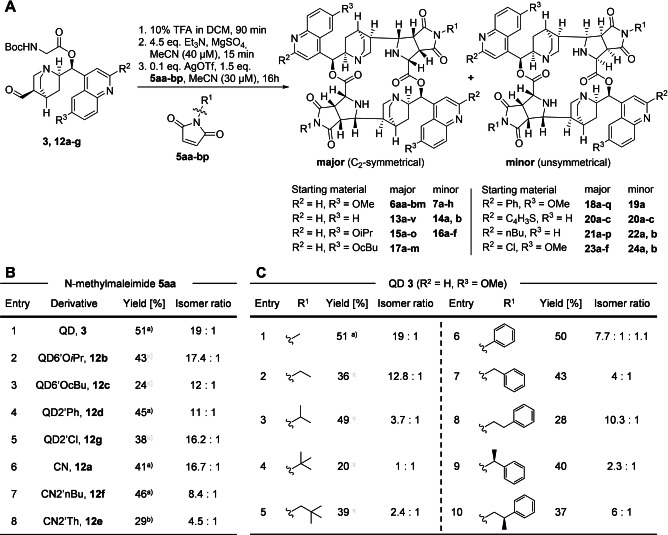
Stereoselective synthesis of homodimeric macrocyclic pseudo‐NPs by 1,3‐dipolar cycloaddition. A) One‐pot three‐step reaction sequence for macrocycle generation; B) Diastereomeric ratios observed for reactions of *N*‐methyl maleimide with different starting materials; C) Diastereomeric ratios observed for reactions of starting material **3** with differently *N*‐substituted maleimides. [a] 3 eq. Et_3_N; [b] 1 : 1 MeCN:DMF as solvent.

In the presence of base and AgOTf as catalyst, azomethine ylides derived from glycine ester Schiff bases underwent dipolar cycloaddition with different maleimides to yield diastereomeric macrocyclic PNPs with varying diastereomeric ratios and in yields of 10–51 % (Figure 1A). Acyclic dipolarophiles were tolerated as well but yielded complex mixtures of diastereomers. Maleimides, however, gave mostly only two distinct and separable products (see Supporting Figure 2).

Ylides derived from α‐substituted amino acids instead of glycine were not tolerated in the cycloaddition. If instead of quinidine, its naturally occurring diastereomer quinine was employed, a mixture of linear and cyclic oligomers was obtained (see Supporting Figure 3).

Substantial efforts were undertaken to optimize the reaction conditions at different stages of the overall sequence, including variation of metal catalyst, solvent and ligands for the metal (see Supporting Information). Notably, addition of mono‐ or bidentate chiral or achiral ligands did lead to a significant drop in yield and did not improve the stereoselectivity, which suggests that the diastereoselectivity observed in the formation of the eight stereocenters originates from substrate control (Figure 1B and 1C).

A wide range of dipolarophiles is accepted in the 1,3‐dipolar cycloaddition (Supporting Figure 2). However, only maleimides react with synthetically viable regio‐ and stereoselectivity. In these cases, the nature of the N‐substituent largely determines the stereoselectivity and the yield (compare Figure 1B and C). For small substituents like methyl groups diastereomeric ratios are high, but introduction of larger substituents, with steric bulk in direct proximity to the maleimide nitrogen, leads to lower diastereoselectivity (Figure 1C, compare entries 1 to 5 and 6 to 10). For some N‐phenyl maleimides, formation of additional diastereomers was observed. If the phenyl ring is in benzylic or homobenzylic position, selectivity is higher and no additional diastereomers were detected (Figure 1C, entries 6 and 7). In depth investigation of these additional diastereomers was not pursued.

### Structure Determination

The fact that only two out of a large number of possible stereoisomers are formed in the two consecutive cycloadditions is remarkable. Combinatorial calculation considering facial selectivity, *endo*/*exo*‐orientation, shape of the azomethine ylides and the fact that the dipoles are dimeric suggests, that a total of 136 diastereomeric 20‐membered macrocycles could, in principle, be formed, yet mainly only two were observed (see the Supporting Information for the calculation).

Given this multitude of possibilities, we attempted to unambiguously determine the structure of the stereoisomers by means of crystal structure analysis. After established crystallization procedures for small molecules had failed, we applied methodology typically employed in protein crystallization. In short, 100 nL aqueous compound solutions were mixed with equal amounts of commercial crystallization buffers via a pipetting robot and automatically imaged periodically, allowing to screen conditions in the thousands (for further details see the Supporting Information).

**Figure 2:**
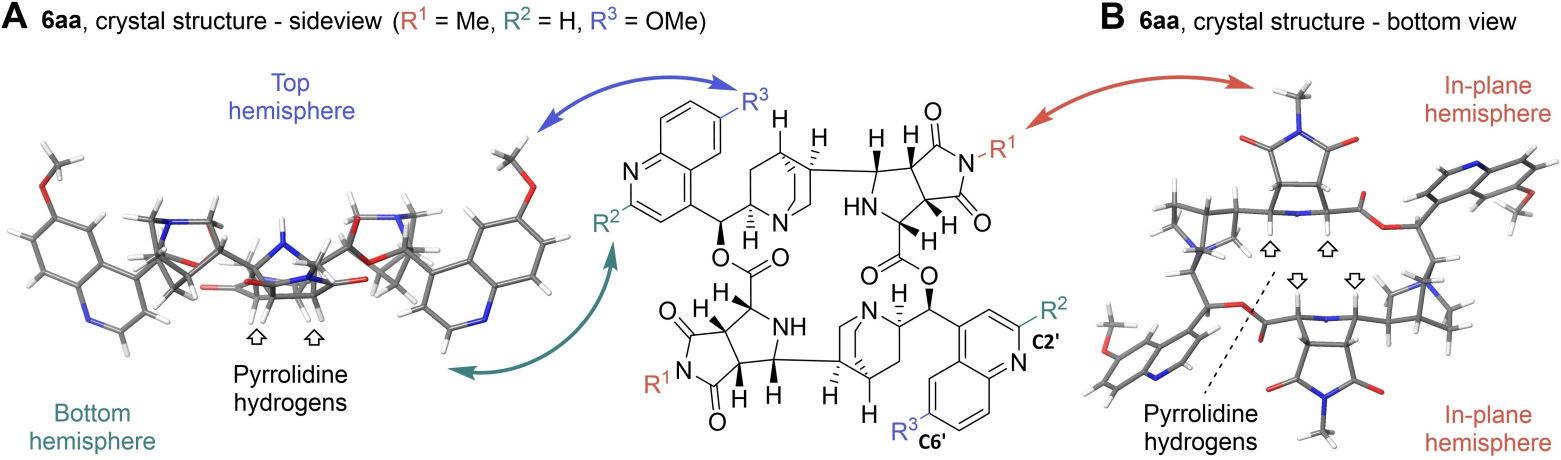
Determination of the absolute configuration of symmetrical **6 aa** via crystal structure and positioning of substituents relative to the plane of the macrocycle (middle). A) Sideview of **6 aa**. B) Bottom view of **6 aa**.

By means of this methodology two structures of symmetrical *bis*‐cycloadducts could be determined, namely the macrocycles **6 aa** and **6 ab** obtained from reactions of QD (**3**) with *N*‐methyl maleimide or *N*‐phenyl maleimide respectively (Figure 2 and Figure 5D respectively).^[31]^

The crystal structures revealed, that this symmetrical diastereomer was formed via a dual *endo*‐1 approach of the dipolarophile, resulting in the all‐*cis* configuration of the annulated pyrrolidine‐hydrogens, that all point down towards the bottom hemisphere of the macrocycle (Figure 2).

**Figure 3:**
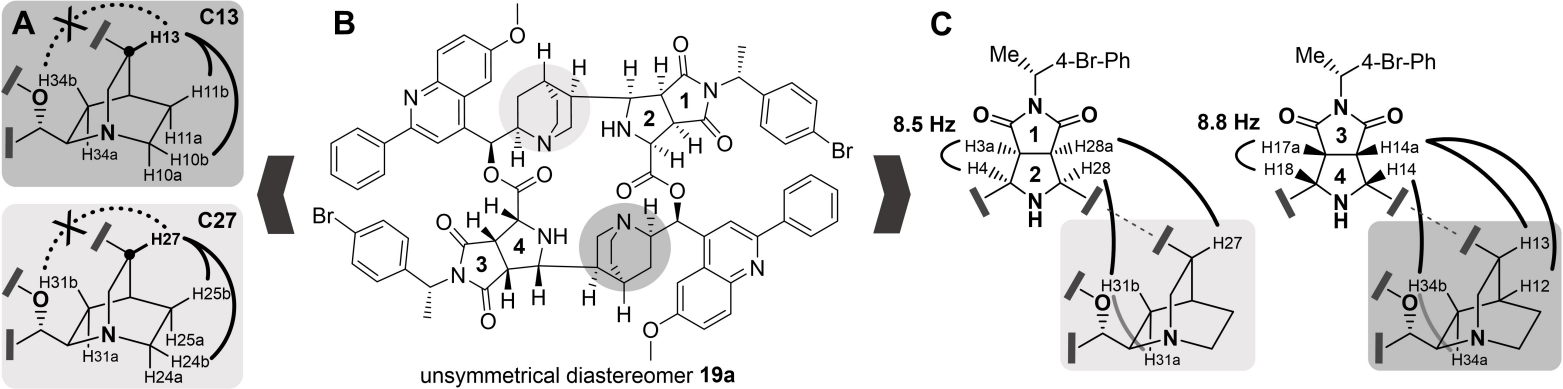
Key NOESY and tROESY interactions in the unsymmetrical diastereomer **19 a** lead to the proposed configuration of the unsymmetrical diastereomer. A) Determining interactions within the quinuclidines for the configurations on C13/C27. B) **19 a** in the proposed configuration. C) Determining interactions within the pyrrolidine hydrogens and between pyrrolidine and quinuclidine hydrogens. Black=NOE/ROE interaction; light grey=passed‐through tROESY interaction with negative sign; thin black arch=indicative coupling constant.

In order to gain insight into the structure of the unsymmetrical diastereomer, which could not be crystallized, we subjected compound **19 a** obtained from the reaction of QD2′Ph (**12 d**) with *N*‐(R)‐1‐(4‐bromophenyl)ethyl maleimide (**5 ac**) to extensive analysis by means of different NMR‐spectroscopic methods (Figure 3 and Supporting Information). As opposed to the symmetrical macrocycle, almost all proton signals of the two different hemispheres of the unsymmetrical macrocycle display differing chemical shifts and could be assigned to either one hemisphere by means of the HMBC technique. Further analysis employing NOESY‐ and tROESY methods and taking different coupling constants into account, led to the following conclusions:

1. NOESY and tROESY couplings within the quinuclidine ring systems (H13 to H10b/H11b but not to H34b; H27 to H24b/H25b but not to H31b), revealed the configuration of C13/C27 as R and thus to be the same as in the symmetrical product (Figure 3A)
2. The coupling constants of the pyrrolidine protons H4 and H18 of 8.5 and 8.8 Hz, respectively, match the symmetrical diastereomer (8.4 Hz). Furthermore, coupling constants in the range of 7–9 Hz are typical for *endo*‐adducts of 1,3‐dipolar cycloadditions of azomethine ylides with maleimides.^[23]^ Thus, these protons should be in *cis*‐arrangement to neighboring H3a/H28a and H17a/H14a (Figure 3C)
3. The observed strong NOE‐ and ROE‐interactions of H14 to H34b and H28 to H31b should exclude *endo* adducts from S‐shaped ylides, that would leave H14 or H28 *trans* to the rest of the pyrrolidine hydrogens (Figure 3C). These interactions could also be found in the spectra of the symmetrical diastereomer.

Collectively, these findings suggest that in the unsymmetrical diastereomer all protons within the different pyrrolidine rings are in *cis*‐arrangement and that, hence, this product can be described as an *endo*‐1/*endo*‐2 adduct (i.e. the two *endo*‐cycloadditions occurred from different faces of the macrocyclic ylides).

**Figure 4:**
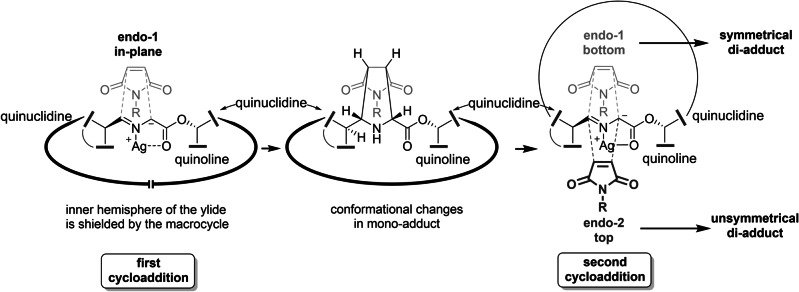
Possible transition states involved in the formation of the dual cycloadducts.

The lack of an additional symmetrical *endo*‐2/*endo*‐2 adduct suggests, that the first 1,3‐dipolar cycloaddition might proceed with a high *endo*‐1‐selectivity. Subsequently, depending on the steric demand and possible conformational changes induced by the first succinimide substituent, the second cycloaddition may proceed via either, an *endo*‐1 (bottom approach) or an *endo*‐2 (top approach) transition state (Figure 4). A strong preference for *endo*‐adducts in general, has been reported for the 1,3‐dipolar cycloaddition of azomethines before.[^20^, ^32^]

This observed selective formation of *endo*‐1/*endo*‐1 and *endo*‐1/*endo*‐2 products can be explained by the macrocyclic nature of the two distinct ylide species involved in the dual cycloaddition, i.e. bis‐imine **4** and the mono‐cycloaddition‐adduct (Figure 4). In bis‐imine macrocycle **4**, one face of the ylide might be pointing into the macrocycle, which would shield this face from approach of the first maleimide. Such a conformation has also been suggested for related di‐adducts synthesized by Rowan and Sanders (see above).^[17]^ The macrocycle formed in the first cycloaddition might adopt a different conformation and thus enable the approach of the second dipolarophile from both opposing faces of the remaining ylide (Figure 4). Large succinimide substituents, such as *tert*‐butyl, may lead to a more pronounced conformational change, resulting in lower facial selectivity in the second cycloaddition (compare Figure 1C entries 1–5 and Figure 4).

**Figure 5:**
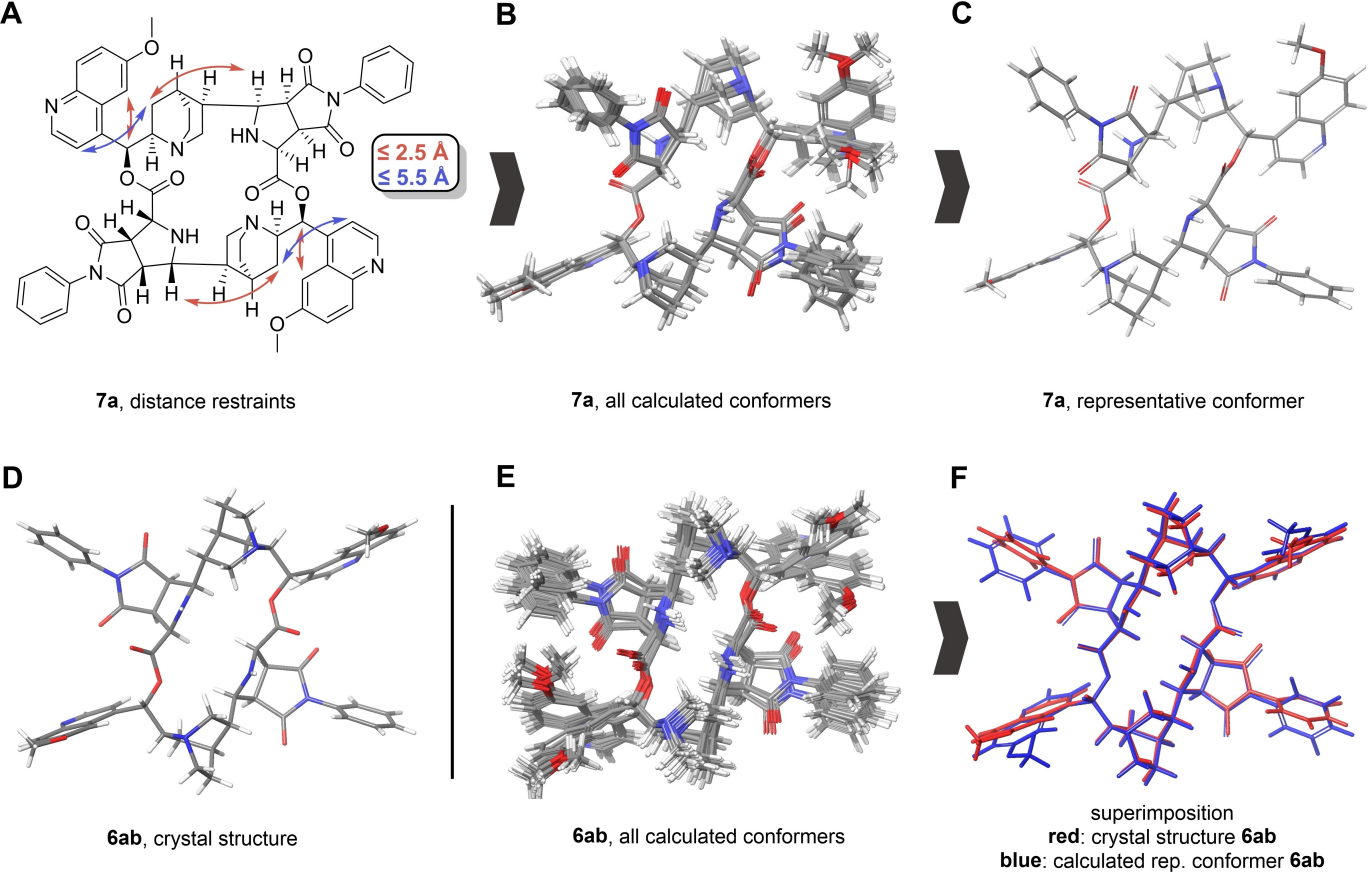
Schematic workflow for the calculation of a representative conformer of unsymmetrical cycloadduct **7 a**. A) Distance restraints derived from NOE‐data of **7 a**, red arrows represent a distance constraint of 2±0.5 Å, blue arrows represent a distance constraint of 3.5±2.0 Å. B) Superimposition of all energy‐optimized conformers of **7 a**. C) Selected representative structure of the largest conformer cluster of **7 a**. D) Crystal structure of symmetrical **6 ab**. E) Analogously produced conformers for **6 ab**. F) Superimposition of a representative conformer of the largest conformer cluster of **6 ab** with the corresponding crystal structure shows the feasibility of the chosen modeling approach for these macrocycles.

To test the plausibility of the proposed configuration and gain further insight into the preferred conformations adopted by the unsymmetrical diastereomers in solution, we sampled the conformational space of minor isomer **7 a** using the conformational sampling algorithm in Maestro MacroModel (OPLS3e force field, H_2_O; version 12.8, Schrodinger). A minimal number of key constraints was set to ensure that the conformational exploration would yield structures in agreement with the NOE data and yet guarantee sufficient rotational freedom. Upper limits of 2.5 Å for the strong interactions (H14 to H34b and H28 to H31b, H5′ to H7 and H5′′ to H21) and 5.5 Å for weaker NOE/ROE signals (H3′ to H34b and H3′′ to H31b) were set. Each of the calculated conformers was subsequently subjected to an energy minimization with the same forcefield. The resulting conformers were clustered via RMSD on all atoms. A representative of the most populated cluster was selected and regarded as the conformation that the molecule adopts in solution most of the time (Figure 5C).^[13]^ For comparison, the same calculations were carried out for the symmetrical diastereomer **6 ab** (Figure 5E), using the same distance restraints. Superimposition of the calculated conformer of **6 ab** with its corresponding crystal structure (Figure 5F) showed, that the chosen in‐silico workflow could accurately predicted the experimentally observed conformation (Figure 5D).

The calculated conformation for the unsymmetrical diastereomer **7 a** underlines the plausibility of the NMR‐derived configuration that was used as an input. Furthermore, the modelled distances within the molecule fit the observed NOE/ROE data well. It revealed an opposite orientation of key functional groups, such as the secondary amine of the pyrrolidines, compared to the symmetrical macrocycle (compare Figure 5C and 5D, further details can be found in the Supporting Information).

### Synthesis of a Compound Collection

The crystal structure and NMR spectroscopic analysis described above indicated that variation of only three positions (six after dimerization) would suffice to install substituents in almost all spacial hemispheres of the macrocycles (R^1^/R^2^/R^3^, Figure 2). Due to the perpendicularity of the quinoline ring of the cinchona alkaloid fragment towards the plane of the macrocycle, C6′ substituents are positioned in the top hemisphere and C2′ substituents in the bottom hemisphere. Succinimide substituents reside in the in‐plane hemispheres. In the minor unsymmetrical diastereomer the situation is comparable, as is evident from inspection of the major conformer identified by means of the structural investigations detailed above (compare Supporting Figure S12).

**Scheme 3:**
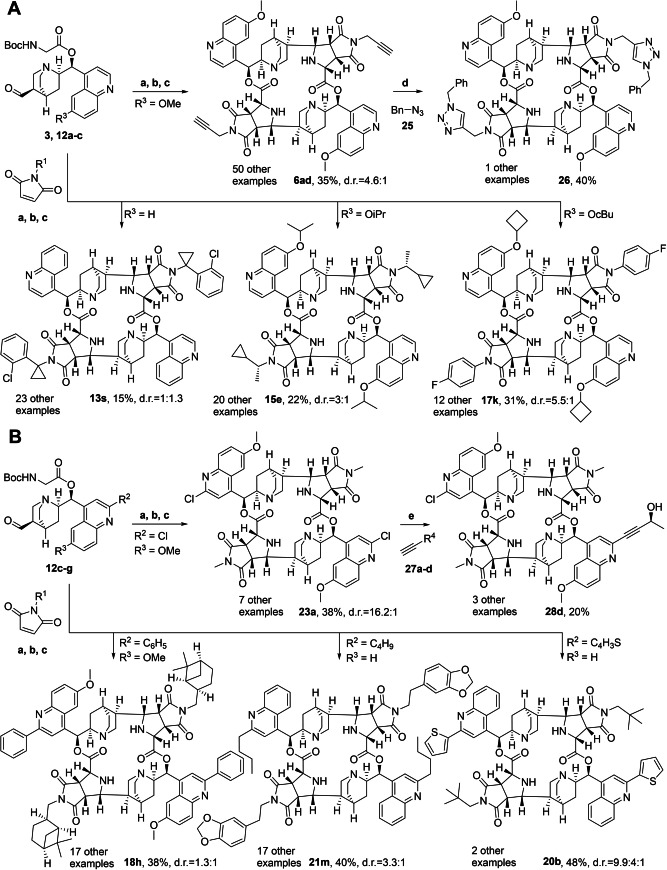
Synthesis of representative, symmetrical members of the compound collection. A) Macrocycles with varying succinimide substitution bearing C6′ quinoline modifications. B) Macrocycles with varying succinimide substitution bearing C2′ and C6′ quinoline modifications. a, b, c) Macrocyclization reaction sequence outlined in Figure 1; d) benzylazide, CuI, K_2_CO_3_, (23–40 %); e) Pd[3,5‐(CF_3_)_2_C_6_H_3_]_3_, terminal alkyne, K_2_CO_3_, Et_3_N, (20–56 %).

In agreement with this reasoning, for the synthesis of a structurally diversified compound collection we introduced different substituents into the building blocks, including reactive handles to enable further chemical derivatization of the macrocycles (e.g. **6 ad** and **23 a**, Scheme 3). Thus, the C2′‐position of quinidine and cinchonine were directly alkylated via the nucleophilic addition of alkyllithium reagents. The resulting dihydroquinolines were then re‐oxidized using manganese dioxide (**10 c**–**e**, Scheme 2).^[27]^ Chlorination in this position was achieved by first generating the quinoline *N*‐oxide by treatment with *m*CPBA, followed by selective reduction of the parallelly formed quinuclidine *N*‐oxide using H_2_SO_3_. Subsequent treatment with POCl_3_ then gave the desired ortho‐chloride (**10 f**, Scheme 2).^[28]^ C6′ ether derivatives were generated from quinidine via boron tribromide mediated demethylation followed by an alkylation with alkyl halides (**10 a** and **10 b**, Scheme 2).^[29]^ Maleimides **5 ad**–**bb** were synthesized from the respective enantiopure primary amines and maleic anhydride (Supporting Information).^[33]^

The chlorine substituent in the C2′‐position of the quinidine enabled functionalization of the macrocycles by means of Sonogashira couplings. The Sonogashira coupling could also be employed to desymmetrize symmetrical macrocycles, when only one equivalent of alkyne was used (e.g. **28 d**, Scheme 3B).

In addition, a propargyl group was introduced through the maleimide employing slightly modified reaction conditions. The terminal alkyne then enabled further modification via Cu^I^‐mediated dipolar cycloadditions with benzyl azide (**26**, Scheme 3A).

Thus, the macrocycles themselves can serve as platforms for further derivatization when suitable reactive handles are introduced.

In total a collection of 163 macrocyclic PNPs was synthesized by means of the short reaction sequences described above. The synthesis of a representative selection of symmetrical macrocycles is shown in Scheme 3.

### Induction of LC3 Lipidation

The macrocyclic PNP‐collection was investigated for bioactivity in phenotypic assays monitoring different biological processes, for instance, Hedgehog pathway activity, bone morphogenetic protein (BMP) signaling, Indoleamine 2,3‐dioxygenase 1 (IDO1) activity and autophagy modulation. Interestingly, the autophagy assay revealed that several members of the collection induced a substantial upregulation of LC3‐lipidation. LC3 is a member of the ubiquitin‐like ATG8 protein family that is required for autophagosome formation during macroautophagy.[^34^, ^35^] During autophagy activation cytosolic LC3 (LC3‐I) is conjugated to the lipid phosphatidylethanolamine to form membrane‐bound LC3‐II which is attached to nascent autophagic structures (e.g. autophagosomes). However, LC3‐lipidation has also been linked to several other biological pathways such as LC3‐associated phagocytosis (LAP)^[36]^ and entosis^[37]^ and is hence not exclusive to macroautophagy activation.^[38]^ Therefore, the image‐based assay using cells expressing EGFP‐LC3 may enable the identification of modulators of new pathways involved in LC3‐associated processes.[^39^, ^40^]

Formation of EGFP‐LC3 punctate structures correlates with lipidation of LC3 and, thereby, association with membranes. EGFP‐LC3 puncta formation was monitored in cells under fed conditions (minimal essential media, MEM). In a separate experiment, the autophagosome‐lysosome fusion inhibitor chloroquine (CQ) was included for inhibition of autophagic flux, leading to the accumulation of EGFP‐LC3 puncta and lipidated LC3.^[41]^

**Figure 6:**
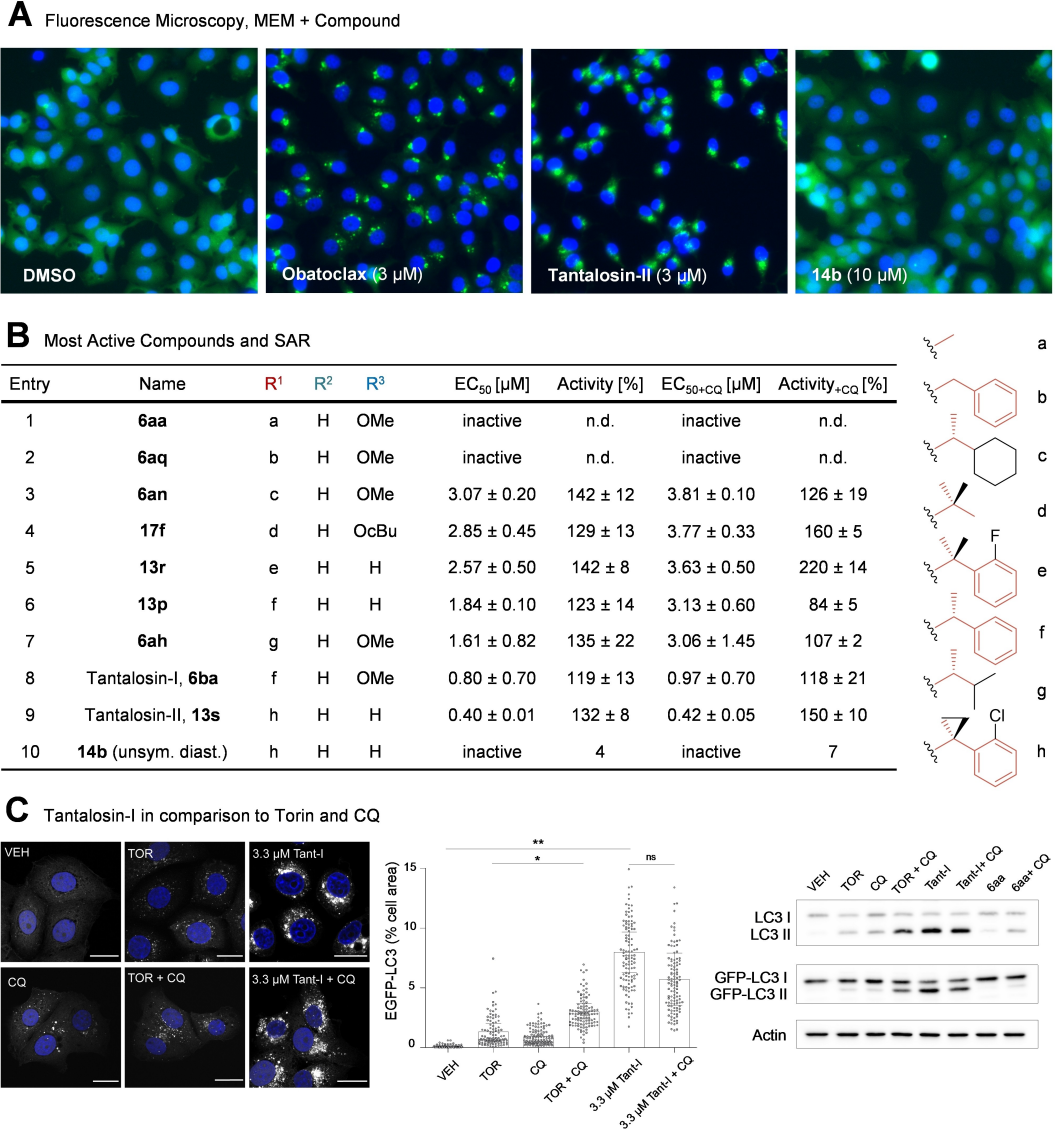
Identification and refinement of an autophagy activation phenotype produced by cinchona macrocycle pseudo‐NPs. A) Detection of EGFP‐LC3 in MCF7‐cells under fed conditions (MEM) 3 h after compound treatment with: DMSO (vehicle control), obatoclax (reference), Tantalosin‐II and its inactive diastereomer **14 b**. Representative images for N=3 are shown. B) The seven most potent inducers of the autophagy activation phenotype relative to obatoclax, as well as three examples for inactive compounds. Mean EC_50_±SD and %Activity±SD each relative to obatoclax (N=3) for MEM and MEM+chloroquine (CQ). C) Confirmation of initial assay data through high‐resolution confocal fluorescence microscopy and western blotting. Representative images of EGFP‐LC3 puncta (left panel). Corresponding quantification: data points represent individual cells (N>30 cells per experiment, *n*=3 pooled); bars are mean±SD for *n*=3; * *p*<0.5, ** *p*<0.05, unpaired t‐test, two‐tailed (middle panel); western blotting using anti‐LC3 and anti‐GFP antibodies *n*=3, representative image is shown (right panel). All compound treatments were performed for 4 hours.

The assay revealed that several symmetrical cinchona‐derived PNPs, such as compound **6 ba** and compound **13 s** (ultimately termed Tantalosin‐I and ‐II, named after the ever‐hungry Tantalus of Greek mythology) induce pronounced EGFP‐LC3 puncta formation with or without CQ co‐treatment (Figure 6A and Figure 6B). In contrast to Torin‐1 (TOR), a known activator of macroautophagy, Tantalosin‐I (Tant‐I) did not induce further increase of EGFP‐LC3 puncta and LC3‐lipidation (Figure 6C). In order to quantify this CQ‐insensitive LC3‐lipidation, the prodigiosin‐derivative obatoclax was employed as reference compound. Obatoclax has been shown to induce LC3 lipidation and to inhibit autophagosome turnover, allowing quantification independent of CQ (Figure 6A).[^42^, ^43^, ^44^] EC_50_‐ and % activity values were calculated relative to the level of EGFP‐LC3 puncta formation in the presence of 3 μM obatoclax (Figure 6B).

With almost exclusively unsubstituted‐ or C6′‐methoxy derivatives among the top scoring compounds, quinoline substitutions (R^2^/R^3^) can be considered to have an overall negative effect on the activity (Figure 6B). Succinimide substituents show a more pronounced influence on the activity. For instance, removal of one methyl group from the initial hit compound Tantalosin‐I, yielded inactive benzyl‐derivative **6 aq** (compare entries 2 and 8, Figure 6B). Keeping the α‐substituted ethyl group, consistently afforded potent derivatives (Figure 6B, R^1^‐substituents c‐h). The most active compound Tantalosin‐II (**13 s**) falls into this category. Its *N*‐substituent was designed to compensate for the α‐stereocenter of Tantalosin‐I via 1,5‐steric repulsion (entry 9, Figure 6B).^[45]^ No unsymmetrical diastereomer showed an induction of LC3‐lipidation, as is exemplified by the Tantalosin‐II diastereomer **14 b** (Figure 6A and Figure 6B, compare entries 9 and 10).

The results of the high‐content assay were verified by high‐resolution confocal fluorescent microscopy using MCF7 cells stably expressing EGFP‐LC3 (Figure 6C). Induction of macroautophagy using the mTOR inhibitor Torin‐1 resulted in an increase in the number of LC3 puncta which was further elevated by CQ‐mediated flux inhibition. In line with the results of high‐content assay, we observed significant EGFP‐LC3 puncta accumulation following Tantalosin‐I treatment which was insensitive to CQ. LC3‐lipidation status was further confirmed by western blotting. Induction of macroautophagy via Torin‐1 resulted in mild elevation of LC3‐II levels. As expected, LC3‐lipidation was higher upon co‐treatment with Torin‐1 and CQ. In agreement with the data of image‐based assay, Tantalosin‐I treatment resulted in a significant accumulation of lipidated LC3 with or without CQ co‐treatment. Compound **6 aa**, an inactive member of the compound collection, did not influence LC3‐lipidation.

These results demonstrate that Tantalosin‐I induces LC3‐lipidation with a phenotype different from well‐established autophagy inducers like Torin‐1. Hence, the macrocycles reported herein can be considered as novel inducers of LC3‐lipidation. Given the manifold roles of LC3 in diverse biological processes, these compounds may provide invaluable tools for the study of LC3‐associated processes.

## Conlusion

In conclusion, we have developed a new class of macrocyclic PNPs which combine different cinchona alkaloid‐inspired fragments in unprecedented arrangements. The macrocyclic PNPs are readily accessible in an efficient one‐pot synthesis which yields the 20‐membered macrocycles incorporating 18 stereocenters with high selectivity and viable yields. Biological analysis of this new PNP‐class yielded a novel class of inducers of LC3‐lipidation which could potentially be employed as invaluable tools to gain further insight into biology. Our results demonstrate that design and synthesis of novel PNPs may yield novel bioactive chemical matter with unexpected and unusual bioactivity, thereby enabling a wider coverage of biologically relevant chemical and biological space.

## Conflict of interest

The authors declare no conflict of interest.

1

## Data Availability

The data that support the findings of this study are available from the corresponding author upon reasonable request.
